# Sensory and Motor Median Nerve Neuropathy Due to a Deep Giant Hand Lipoma: A Case Report

**DOI:** 10.1055/s-0041-1726072

**Published:** 2021-04-19

**Authors:** Ioannis M. Stavrakakis, Vasiliki Georgopoulou, George E. Magarakis, Maria S. Katsafarou

**Affiliations:** 1 Departamento de Ortopedia, General Hospital of Agios Nikolaos, Creta, Grécia; 2 Departamento de Ortopedia, Venizeleio General Hospital of Heraklion, Creta, Grécia

**Keywords:** carpal tunnel syndrome, hand, lipoma, soft tissue neoplasms, median nerve neuropathy

## Abstract

Lipomas are the most common soft-tissue tumors in the human body, but their location in the hand is rare. Symptomatic hand lipomas, due to nerve compression, are even rarer. We present a case of median nerve neuropathy as a result of a giant palm lipoma, located on the thenar and hypothenar areas of the hand. The patient had typical symptoms of carpal tunnel syndrome, along with compromised thumb motion. Intraoperatively, the recurrent motor branch of the median nerve was sitting on the lipoma under a great tension. This particular location of the motor branch of the median nerve in relation to the lipoma makes this case unique. The tumor was excised protecting the neurovascular structures, and a few weeks later the patient regained full thumb motion, grip strength, and resolution of dysesthesia.

## Introduction

Hand lipomas represent ∼ 15% of all soft-tissue tumors in the body. 1 Their occurrence in the hand is rare, accounting to 1 to 3.8% of all benign hand soft-tissue tumors. 2 Their etiology remains unclear, but there is evidence supporting that either genetic, traumatic, or metabolic factors can cause them. 1 3 They are mainly asymptomatic, but neuropathy might develop, due to nerve compression, with subsequent pain and functional disability. 4 The main indications for surgical excision are pain, compromised function, cosmetic reasons, and large size. 5 We present the case of a female patient, who presented with a soft, enlarged mass on her right palm. She also described symptoms compatible with carpal tunnel syndrome, as well as compromised thumb movements. The patient was successfully treated with tumor excision. This case highlights a rare location of the recurrent motor branch of the median nerve in contact to a hand lipoma. Appropriate intraoperative figures are provided as well, to present this rare image adequately. This case presentation was performed in compliance with the world medical association of Helsinki on ethical principles for medical research involving human subjects and was reviewed by the Institutional Review Board.

## Case Presentation

A 49-year-old female patient presented to our institution reporting a relatively large mass over her right palm. The patient noticed its presence 6 months prior to the appointment. She mentioned that this mass had gotten progressively larger over the last 3 months. During the same period, she developed tingling on the three radial digits, as well as compromised motion of her thumb and reduced grip strength. The patient had a few lipomas removed from her skull in the past. Her mother also had the same problem.

**Fig. 1:**
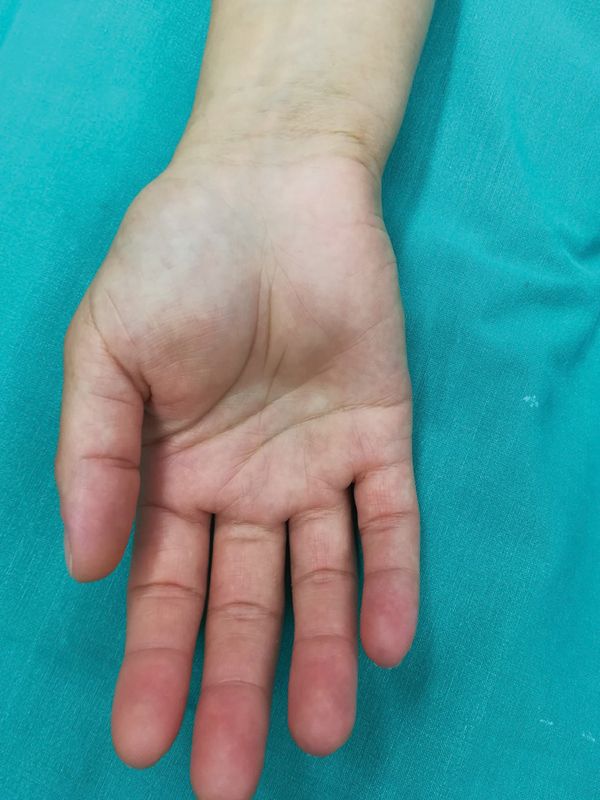
Clinical Image of the patient's right palm. A soft-tissue mass of the thenar and hypothenar area is visible.

On examination, a soft, firm, mildly tender mass was located on the mid palm, extending from the thenar to the hypothenar area ( Fig. 1 ). Tumor's percussion reproduced tingling on the radial three digits, indicating a strongly positive Tinel sign. Palmar abduction of the thumb was weak as well. No thenar atrophy was identified, though, probably because patient's symptoms were not present for a long time. Carpal tunnel syndrome associated to median nerve compression caused by a hand lipoma was suspected. The patient underwent electromyography (EMG), which confirmed median nerve neuropathy. Magnetic resonance imaging (MRI) revealed a deep 5,7 × 2,4 × 2 cm palmar lipoma, and surgical excision was scheduled.

**Fig. 2:**
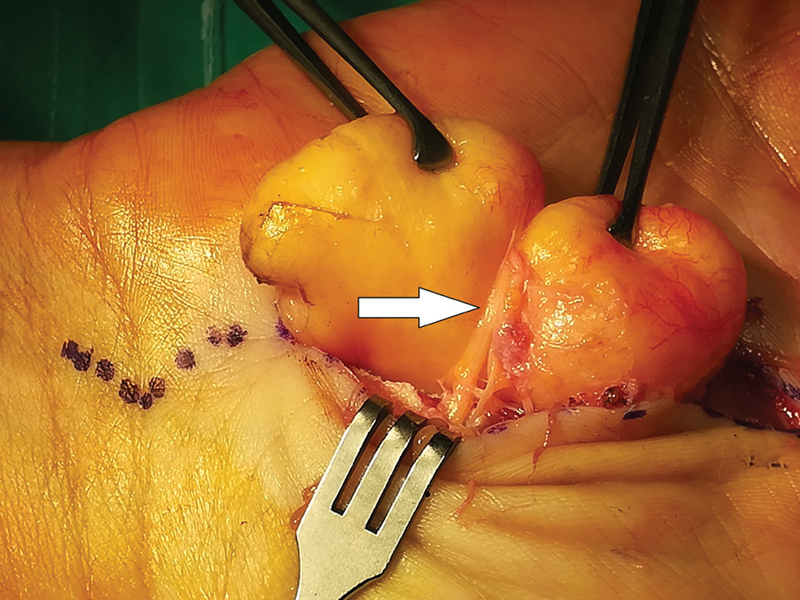
Intraoperative view showing the recurrent motor branch of the median nerve (white arrow), sitting between the lipoma's two lobes. The nerve is obviously under tension.

**Fig. 3:**
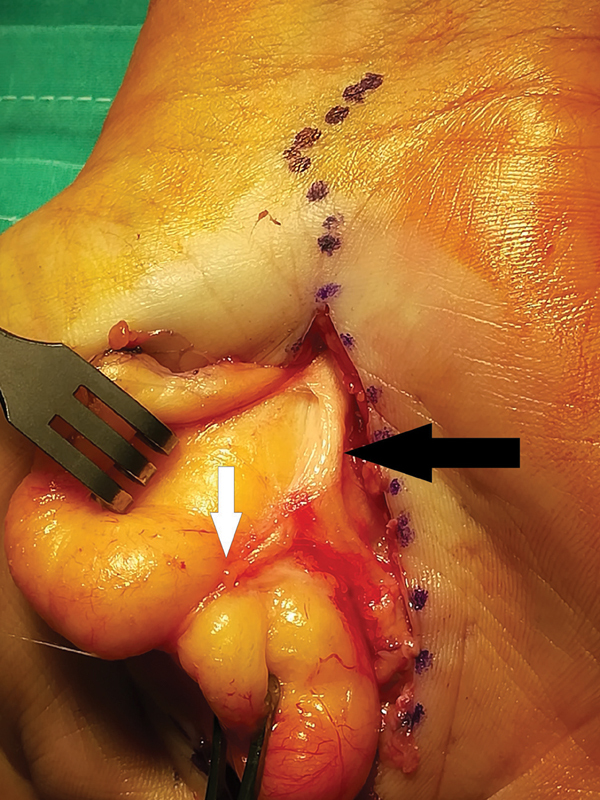
Intraoperative view after releasing the distal part of the carpal tunnel. The median nerve (black arrow) and its motor branch (white arrow) are lying onto the tumor.

**Fig. 4:**
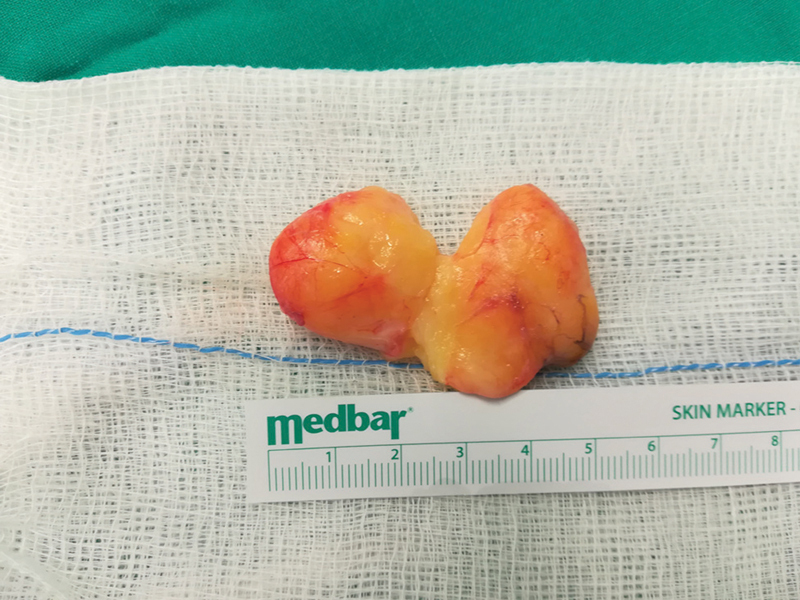
Excised lipoma. It had to be dissected into two parts, to avoid damaging the motor branch of the median nerve. The two pieces are approximated in this photo.

**Fig. 5:**
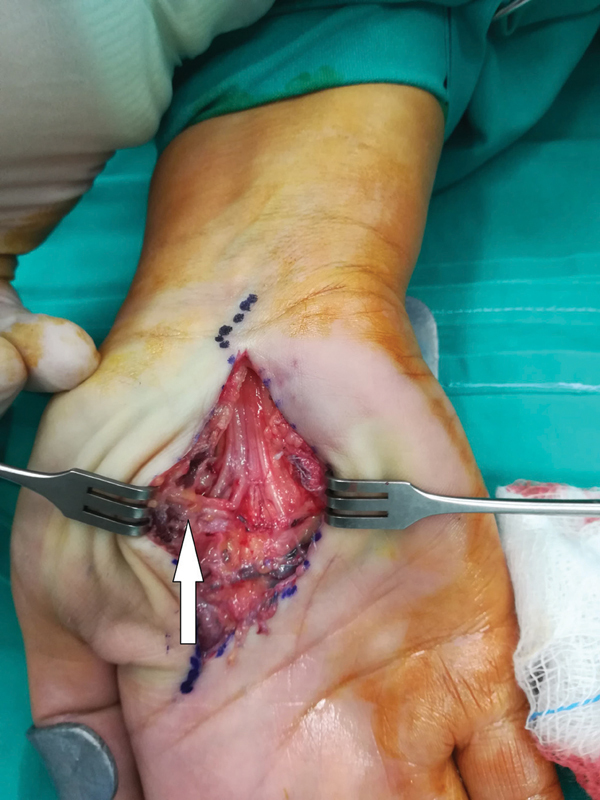
View of the palm after lipoma excision. White arrow is pointing at the recurrent motor branch of the median nerve.

Under general anesthesia and humeral tourniquet application, a midline longitudinal volar incision, extending from the wrist crease all the way distally across the “lifeline,” was performed. A large bilobulated yellowish tumor was identified between the palmar aponeurosis and underlying flexor tendons, occupying the thenar and hypothenar areas. It originated from the distal part of the carpal tunnel in close proximity to the median nerve. The recurrent motor branch of the median nerve was found to be under great tension, on the lipoma, and it was literally splitting the tumor into two lobes ( Figs. 2 , 3 ). We believe that this intraoperative image is unique. The lipoma had to be dissected for it to be removed safely, without damaging the nerve ( Fig. 4 ). The carpal tunnel was also released to achieve an adequate decompression of the median nerve ( Fig. 5 ).

The wound healed uneventfully and 3 months later, the patient reported complete resolution of dysesthesia and improved thumb motion. It is though noticed mild stiffness of the fingers, probably due to some form of algodystrophy, which is currently managed with physiotherapy.

## Discussion

Lipomas, although common benign tumors, rarely present in the hand. They are predominantly superficial and asymptomatic. Hand lipomas located in the deep palmar space are more infrequent. 6 Their etiology is multifactorial. Traumatic, genetic, and metabolic factors have been considered as potential causes. Genetic mutations in the chromosomal region 12q13–15 have been incriminated. 1 6 Our patient reported the excision of multiple lipomas' from her skull in the past, as well as a genetic predisposition, indicating a probable familial multiple lipomatosis. Blunt trauma may lead to a lipoma formation through preadipocyte tissue differentiation, stimulated by inflammatory mediators' release and hematoma creation. Metabolic disorders, such as diabetes, hyperlipidemia, and endocrinopathies have also been related to lipomas. 3

It is widely accepted that tumors larger than 5 cm are considered giant lipomas, 1 2 4 and they present a higher possibility of malignancy. 7 Hand lipomas can be either superficial or deep. Deep lipomas are categorized as endovaginal, if they are located inside the tendon sheath, or epivaginal, if they are located outside of it. 6 8 Depending on their location, hand lipomas can be either asymptomatic or they can produce pain, tingling or motor deficits via compressing adjacent structures, such as nerves or tendons. The former symptoms along with tumor size and cosmetic concerns, are indications for surgery. 3 9

Several cases of median nerve compression have been reported in the literature, mimicking a carpal tunnel syndrome, 1 2 5 6 but very few cases are reported having a compromised thumb motion due to compression of the median nerve's motor branch. 9 Our case involved dysfunction of the thenar muscles, confirmed by EMG studies, in addition to fingers' tingling due to median nerve neuropathy, which was caused by compression of the lipoma. Most lipomas are superficial, and they might compress palmar structures from above, whereas deep lipomas can blend with nerves or tendons in various ways. One can find them passing either above 10 or even through the tumor. 9 Our case represents a very rare image of the recurrent motor branch of the median nerve, which was passing above the tumor, between its two lobes, making the nerve vulnerable during excision.

Surgical excision, when indicated, is the treatment of choice for hand lipomas. Few complications are possible, such as hematoma, and paresis or dysesthesia due to iatrogenic nerve injury. Recurrence is very rare and concerns mainly incompletely resected deep lipomas. 3 Malignant transformation is also extremely rare. 9

## Conclusion

We report a rare case of sensory and motor median nerve neuropathy caused by a deep palmar giant lipoma. The close relation of the recurrent motor branch of the median nerve to the lipoma, which is described in our case, highlights the attention required from the surgeon during surgery to avoid a devastating iatrogenic injury.
