# Supplementary material for: Sensory and Motor Median Nerve Neuropathy Due to a Deep Giant Hand Lipoma: A Case Report
Source: Rev Bras Ortop (Sao Paulo). 2021 Apr 19;59(2):e323–6. doi: 10.1055/s-0041-1726072 (PMC11006529; doi:10.1055/s-0041-1726072)
Supplement: Related texts

# Research article: Neuropatia sensorial e motora do nervo mediano por um lipoma gigante na mão: Relato de caso

**Keywords:** síndrome do túnel do carpo, lipoma, mão, neoplasias de tecido mole, neuropatia do nervo mediano

## Abstract

Os lipomas são os tumores de partes moles mais comuns no corpo humano, mas sua localização na mão é rara. Os lipomas de mão que causam sintomas por compressão do nervo são ainda mais raros. Apresentamos um caso de neuropatia do nervo mediano decorrente de um lipoma palmar gigante, localizado nas regiões tenar e hipotenar da mão. A paciente apresentava sintomas típicos de síndrome do túnel do carpo, além de comprometimento dos movimentos do polegar. Durante a cirurgia, o ramo motor recorrente do nervo mediano repousava sobre o lipoma sob grande tensão. Esta localização particular do ramo motor do nervo mediano em relação ao lipoma torna este caso único. O tumor foi extirpado, protegendo as estruturas neurovasculares e, poucas semanas depois, a paciente havia recuperado totalmente os movimentos do polegar e força de preensão, além de apresentar resolução da disestesia.

## Introdução

Os lipomas de mão representam cerca de 15% de todos os tumores de tecidos moles no corpo. 1 Sua ocorrência na mão é rara, correspondendo entre 1 e 3,8% de todos os tumores benignos de tecidos moles da mão. 2 Sua etiologia ainda é obscura, mas há evidências da participação de fatores genéticos, traumáticos ou metabólicos. 1 3 Em sua maioria, são assintomáticos, mas podem causar neuropatia devido à compressão do nervo, com dor e incapacidade funcional. 4 As principais indicações para a excisão cirúrgica são dor, comprometimento funcional, motivos estéticos e porte extenso. 5 Apresentamos o caso de uma paciente do sexo feminino com uma massa macia e crescente na palma da mão direita. A paciente também relatou sintomas compatíveis com a síndrome do túnel do carpo e comprometimento dos movimentos do polegar. A paciente foi tratada com sucesso por meio da excisão do tumor. Este caso destaca uma localização rara do ramo motor recorrente do nervo mediano em contato com um lipoma de mão. Dados intraoperatórios apropriados também são mostrados para boa caracterização desse quadro raro. Este caso foi apresentado em conformidade com os princípios éticos para pesquisas médicas em seres humanos da Declaração de Helsinque da Associação Médica Mundial e o Comitê de Revisão Institucional.

## Apresentação do Caso

Uma paciente de 49 anos foi atendida em nossa instituição e relatou uma massa relativamente grande na palma da mão direita. A paciente notou sua presença há 6 meses e mencionou que a massa cresceu de maneira progressiva nos últimos 3 meses. Durante o mesmo período, passou a apresentar formigamento nos três dedos radiais, bem como comprometimento dos movimentos do polegar e redução da força de preensão. A paciente foi submetida à remoção de alguns lipomas do crânio no passado. A mãe dela também tinha o mesmo problema.

Ao exame, uma massa macia, firme e com sensibilidade branda foi observada no meio da palma da mão, estendendo-se da área tenar à hipotenar ( Fig. 1 ). A percussão do tumor reproduziu formigamento nos três dígitos radiais, indicando a forte positividade do sinal de Tinel. A abdução palmar do polegar era fraca, mas não havia atrofia tenar, provavelmente devido ao pouco tempo de evolução dos sintomas. Suspeitou-se de síndrome do túnel do carpo associada à compressão do nervo mediano por lipoma na mão. A paciente foi submetida à eletromiografia (EMG), que confirmou a neuropatia do nervo mediano. A ressonância magnética (RM) revelou um lipoma palmar profundo de 5,7 × 2,4 × 2,0 cm e a excisão cirúrgica foi agendada.

**Fig. 1:** Imagem clínica da palma direita da paciente, mostrando uma massa de tecido mole nas áreas tenar e hipotenar.

Sob anestesia geral e com aplicação de torniquete umeral, uma incisão volar longitudinal foi realizada na linha média, estendendo-se da prega do punho distalmente ao longo da “linha da vida”. Um grande tumor bilobulado e amarelado foi identificado entre a aponeurose palmar e os tendões flexores subjacentes, ocupando as áreas tenar e hipotenar. O tumor se originou na parte distal do túnel do carpo, próximo ao nervo mediano. O ramo motor recorrente do nervo mediano estava sob grande tensão, sobre o lipoma, e literalmente dividia o tumor em dois lobos ( Fig. 2 e 3 ). Acreditamos que esta imagem intraoperatória seja única. O lipoma teve que ser dissecado para ser removido com segurança, sem danificar o nervo ( Fig. 4 ). O túnel do carpo também foi abordado para descompressão adequada do nervo mediano ( Fig. 5 ).

**Fig. 2:** Vista intraoperatória do ramo motor recorrente do nervo mediano (seta branca), situado entre os dois lobos do lipoma. O nervo está obviamente sob tensão.

**Fig. 3:** Vista intraoperatória após liberação da parte distal do túnel do carpo. O nervo mediano (seta preta) e seu ramo motor (seta branca) estão situados sobre o tumor.

**Fig. 4:** Lipoma excisado. O tumor teve que ser dissecado em duas partes para evitar lesão do ramo motor do nervo mediano. As duas peças foram aproximadas para esta foto.

**Fig. 5:** Vista da palma após a excisão do lipoma. A seta branca aponta o ramo motor recorrente do nervo mediano.

A ferida cicatrizou sem intercorrências e, 3 meses depois, a paciente relatou resolução completa da disestesia e melhora dos movimentos do polegar. No entanto, apresentava uma leve rigidez dos dedos, provavelmente devido a alguma forma de algodistrofia, atualmente tratada com fisioterapia.

## Discussão

Os lipomas, embora tumores benignos comuns, são raramente observados na mão. São predominantemente superficiais e assintomáticos. Os lipomas de mão localizados no espaço palmar profundo são ainda mais raros. 6 Sua etiologia é multifatorial. Fatores traumáticos, genéticos e metabólicos são considerados possíveis causas. Mutações genéticas na região cromossômica 12q13-15 foram incriminadas. 1 6 Nossa paciente relatou a excisão de múltiplos lipomas do crânio no passado, bem como uma predisposição genética, indicando uma provável lipomatose múltipla familiar. O trauma contuso pode levar à formação de lipoma por meio da diferenciação do tecido pré-adipócito, estimulada pela liberação de mediadores inflamatórios e formação de hematoma. Distúrbios metabólicos, como diabetes, hiperlipidemia e endocrinopatias, também foram relacionados a lipomas. 3

É amplamente aceito que tumores com tamanho superior a 5 cm são considerados lipomas gigantes, 1 2 4 com maior probabilidade de comportamento maligno. 7 Os lipomas da mão podem ser superficiais ou profundos. Os lipomas profundos são classificados como endovaginais, caso localizados dentro da bainha do tendão, ou epivaginais, se estiverem localizados fora dela. 6 8 Dependendo de sua localização, os lipomas de mão podem ser assintomáticos ou causar dor, formigamento ou déficit motor por compressão de estruturas adjacentes, como nervos ou tendões. Os primeiros sintomas, assim como o tamanho do tumor e preocupações estéticas, são indicações para a cirurgia. 3 9

Vários casos de compressão do nervo mediano foram relatados na literatura, simulando a síndrome do túnel do carpo, 1 2 5 6 mas há pouquíssimas descrições de comprometimento do movimento do polegar por compressão do ramo motor do nervo mediano. 9 Nosso caso apresentava disfunção da musculatura tênar, comprovada por estudos de EMG, além de formigamento nos dedos por neuropatia do nervo mediano, que foi provocada pela compressão do lipoma. A maioria dos lipomas é superficial e pode comprimir as estruturas palmares acima, enquanto os lipomas profundos podem se fundir a nervos ou tendões de várias maneiras. Essas estruturas podem repousar acima do tumor 10 ou mesmo atravessá-lo. 9 Nosso caso representa uma imagem muito rara do ramo motor recorrente do nervo mediano, que passava sobre o tumor, entre seus dois lobos, e era vulnerável durante a excisão.

A excisão cirúrgica, quando indicada, é o tratamento de escolha para os lipomas de mão. Poucas complicações são descritas, como hematoma e paresia ou disestesia por lesão nervosa iatrogênica. A recidiva é muito rara e ocorre principalmente em lipomas profundos submetidos à ressecção incompleta. 3 A transformação maligna também é extremamente rara. 9

## Conclusão

Relatamos um caso raro de neuropatia sensorial e motora do nervo mediano causada por um lipoma gigante palmar profundo. A estreita relação do ramo motor recorrente do nervo mediano com o lipoma, descrita em nosso caso, destaca a atenção que o cirurgião deve ter durante o procedimento para evitar uma lesão iatrogênica devastadora.
